# Lessons from the deep: mechanisms behind diversification of eukaryotic protein complexes

**DOI:** 10.1111/brv.12988

**Published:** 2023-06-19

**Authors:** Galina Prokopchuk, Anzhelika Butenko, Joel B. Dacks, Dave Speijer, Mark C. Field, Julius Lukeš

**Affiliations:** ^1^ Institute of Parasitology, Biology Centre, Czech Academy of Sciences Branišovská 1160/31 České Budějovice 37005 Czech Republic; ^2^ Faculty of Science University of South Bohemia Branišovská 1160/31 České Budějovice 37005 Czech Republic; ^3^ Life Science Research Centre, Faculty of Science University of Ostrava Chittussiho 983/10 Ostrava 71000 Czech Republic; ^4^ Division of Infectious Diseases, Department of Medicine University of Alberta 1‐124 Clinical Sciences Building, 11350‐83 Avenue Edmonton T6G 2R3 Alberta Canada; ^5^ Centre for Life's Origins and Evolution, Department of Genetics, Evolution and the Environment University College London Darwin Building, Gower Street London WC1E 6BT UK; ^6^ Medical Biochemistry, Amsterdam UMC University of Amsterdam Meibergdreef 15 Amsterdam 1105 AZ The Netherlands; ^7^ School of Life Sciences University of Dundee Dow Street Dundee DD1 5EH Scotland UK

**Keywords:** molecular evolution, evolutionary mechanisms, gene replacement, constructive neutral evolution, protein complexes, evolutionary divergence

## Abstract

Genetic variation is the major mechanism behind adaptation and evolutionary change. As most proteins operate through interactions with other proteins, changes in protein complex composition and subunit sequence provide potentially new functions. Comparative genomics can reveal expansions, losses and sequence divergence within protein‐coding genes, but *in silico* analysis cannot detect subunit substitutions or replacements of entire protein complexes. Insights into these fundamental evolutionary processes require broad and extensive comparative analyses, from both *in silico* and experimental evidence. Here, we combine data from both approaches and consider the gamut of possible protein complex compositional changes that arise during evolution, citing examples of complete conservation to partial and total replacement by functional analogues. We focus in part on complexes in trypanosomes as they represent one of the better studied non‐animal/non‐fungal lineages, but extend insights across the eukaryotes by extensive comparative genomic analysis. We argue that gene loss plays an important role in diversification of protein complexes and hence enhancement of eukaryotic diversity.

## I. INTRODUCTION

Eukaryogenesis, the transition from prokaryotic to eukaryotic cells, encompassed multiple innovations, with emergence of complex intracellular compartmentalisation being one of the most dramatic. Intracellular organelles are either of endogenous origin, such as the nucleus, flagellum and endomembrane system, or result from endosymbiotic events, exemplified by the mitochondrion and plastids. For endogenous origin compartments, the genes required to support these subcellular structures are derived from ancestral archaeal genes, but are now frequently expanded into large paralogous families (Dacks & Field, 2018; Field & Rout, 2019). Further, a considerable contribution emanated from endosymbiont genomes to support the mitochondrion and plastids (Gray, 2012). Regardless of their ultimate origins, it is generally accepted that the major eukaryotic compartments with the exception of plastids emerged in the period between the first and the last eukaryotic common ancestor (FECA and LECA, respectively) (Martin, Garg & Zimorski, 2015; Speijer, 2020).

**Fig. 1:**
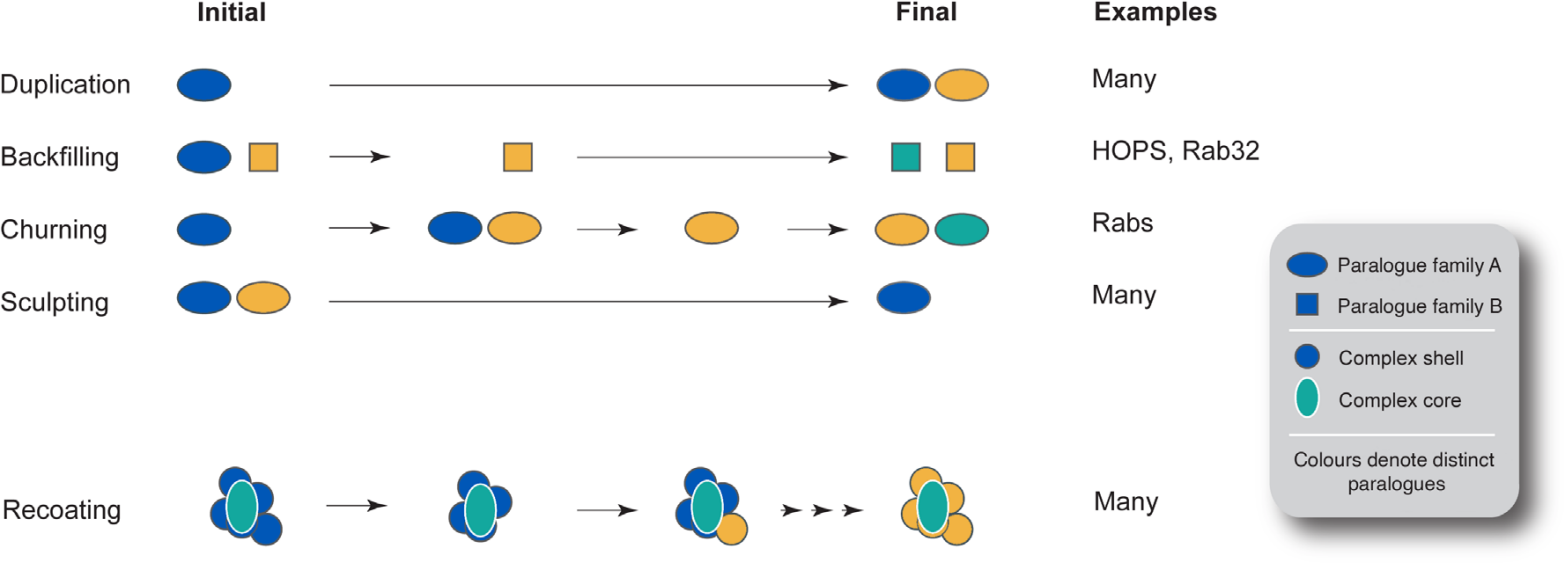
Selected modes of protein complex evolution. Evolution of protein complexes *via* paralogous gene duplication, backfilling, churning, sculpting or recoating. In duplication, a simple event creating a second paralogue allows the evolution of a new function. In backfilling, a lost component is substituted by the expansion of the remaining subunits. For churning, which is a special case of duplication, continual generation of new paralogues coupled with losses serves to diversify a gene family without an apparent change in overall number of paralogues. Sculpting represents a case of simple gene loss. Lastly, recoating describes the process whereby a complex protein core, that is surrounded by an additional protein shell of interactors, sees replacement of the shell but retention of the core subunits. Colours represent different paralogues. HOPS, homotypic fusion and vacuole protein sorting complex; Rabs, Ras‐related in brain proteins.

**Fig. 2:**
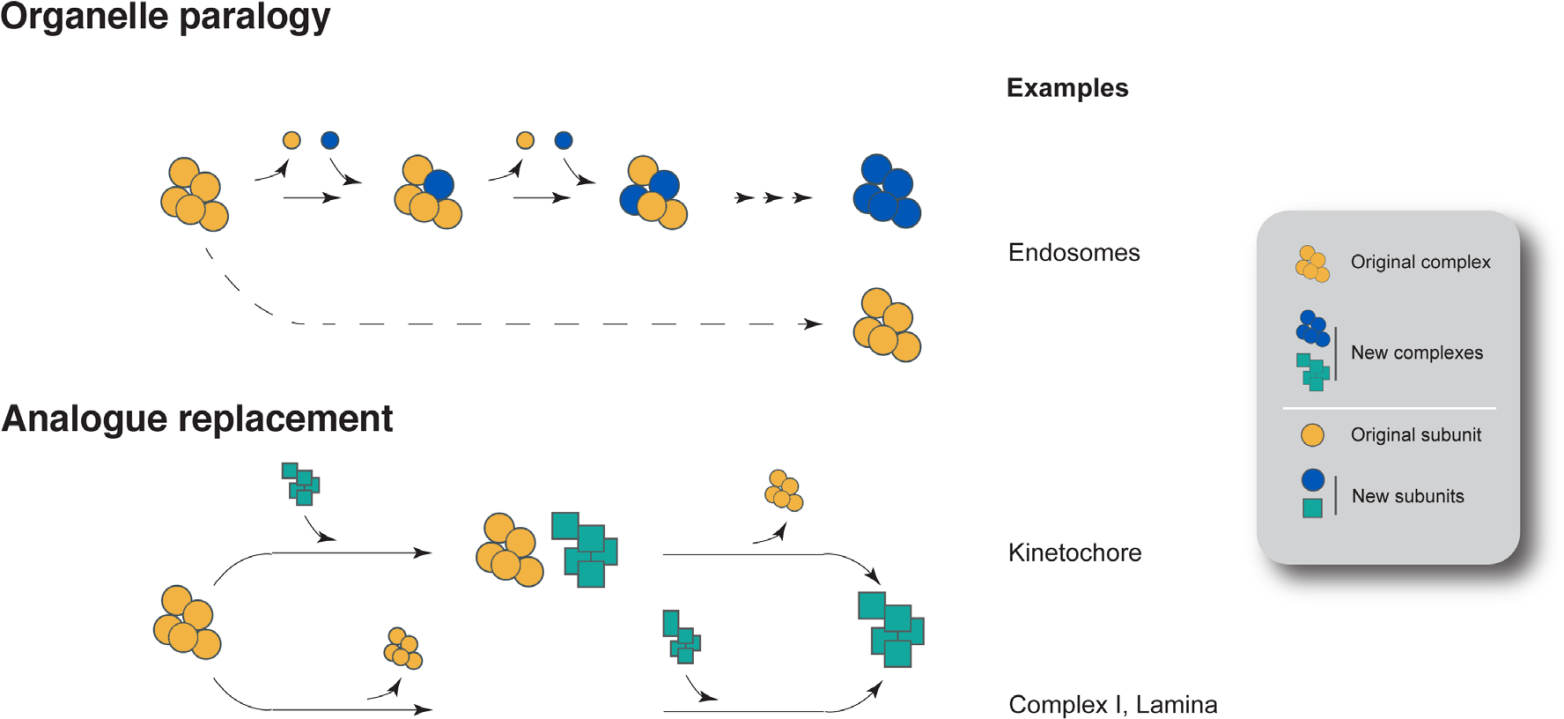
Organelle paralogy and replacement in the evolution of complexes. Top: original organelle paralogy model. Duplication of one paralogue encoding a protein complex subunit allows one of the copies to gain new functions (blue circle), evolving interactions with the original subunits (yellow circles), as well as with new proteins (additional blue circles), these possibly facilitated by mutations and subsequent changes in binding specificity. Importantly, the original complex is retained. Bottom: analogue replacement, a more severe form of complex evolution. In the upper row, exemplified by the evolution of an apparently novel kinetochore in kinetoplastids, the entire complex (or the vast majority) is replaced. We speculate that this took place *via* a neofunctionalisation mechanism, as kinetochore functionality is essential and therefore loss is unlikely to occur. In the lower row, there are clear cases of loss of a system, and its replacement. Respiratory complex I loss is known as are examples of organisms that lack an apparent lamina, *albeit* with some functionality subtended by non‐conventional means. See text for details.

We, and others, have reported that the LECA was likely considerably more complex in terms of the numbers of organelles, protein‐coding capacity and size of many paralogous families than many extant lineages (Koumandou *et al*., 2013; O'Malley, Wideman & Ruiz‐Trillo, 2016; Wideman & Muñoz‐Gómez, 2016). Considering the dynamic nature of eukaryotic genomes post‐LECA, this indicates that gene loss has played a significant role in the evolution of many eukaryotic lineages (Koumandou *et al*., 2013). A number of mechanisms for diversification within paralogous families and protein complexes have been described (Dacks & Field, 2018): (*i*) gene duplication; (*ii*) replacement upon gene loss by recruitment of an unrelated/analogous protein either allowing for, or compensating for, loss of the original protein (backfilling); (*iii*) expansion and contraction of paralogue families (churning); and (*iv*) loss of a gene (sculpting) (Fig. 1). Each of these processes, as well as other possibilities not considered here, can lead to changes in the composition of protein complexes and, in some cases, the evolution of novel complexes (Fig. 2).

The LECA existed at least 10^9^ years BCE (Eme *et al*., 2014) and, assuming a 90‐min generation time (typical for *Saccharomyces cerevisiae*), more than 10^12^ generations might have passed since, allowing a huge variety of forms and lifestyles to arise. Among extant eukaryotes, microbial lineages contained within the supergroups TSAR (Telonemia, Stramenopila, Alveolata, and Rhizaria), Metamonada and Discoba dominate the global biomass and are highly genetically diverse (Katz, 2012). Evolutionary innovations in microorganisms are likely facilitated by both short generation times and population bottlenecks. The latter increases the frequency of fixation of alleles (Wolf & Koonin, 2013), and is a common occurrence in parasitic protists and other potentially isolated populations. Notably, the composition of many protein complexes in protists can approach or even exceed that of multicellular organisms in terms of distinct gene products, demonstrating that protists are, at the cellular level, highly sophisticated and complex organisms (Gray *et al*., 2020). Although large protein complexes are intuitively assumed to have the capacity for more sophisticated functionality (regulation, flexibility, etc.), this may not always be the case. As postulated by Constructive Neutral Evolution (CNE) theory, complexity may increase in the absence of positive selection, hence bringing no selective advantage, at least initially (Stoltzfus, 1999; Lukeš *et al*., 2011). CNE seems to explain some of the diversity of mitochondrial respiratory complexes and protein import machineries. Although additional evidence is needed, the available data indicate that, in these cases, an increase in compositional complexity *per se* does not provide new functions or increased fitness (Muñoz‐Gómez *et al*., 2021). This view is supported by the varying composition of protein complexes from the diverse eukaryotic lineages considered below.

A growing interest in divergent lineages, due to recognition of their importance in multiple contexts, such as ecology and human health, and an increase in genome sequencing, has led to direct experimental characterisation of protein complexes from a taxonomically broad range of organisms. Here, we consider experimental and genomics evidence from organisms across the eukaryotic tree and assess the origins and elaboration of complexes supporting organelle biogenesis and function. We focus on key complexes of the endomembrane system, nucleus, and mitochondrion from multiple organisms, but in particular *Trypanosoma brucei*, a member of the Kinetoplastea (phylum Euglenozoa). Despite being a parasitic organism with somewhat reduced complexity, trypanosomes remain one of the most intensively studied unicellular organisms outside of the opisthokonts. Importantly, this includes direct experimental evidence for a wealth of protein complexes and extends to localisation of the vast majority of proteins encoded by the genome (Tinti & Ferguson, 2022; Billington *et al*., 2023). We compare and discuss some well‐studied protein complexes in eukaryotes based on the available literature, and for the less‐investigated examples we performed additional comparative genomic analyses using a representative (i.e. covering a large swathe of total eukaryotic diversity) set of 23 eukaryotic genomes/transcriptomes. We suggest that many complexes retain a conserved core with, unexpectedly, widely varying peripheral components. In extreme cases, entire complexes are replaced. We also found indications that gene loss has facilitated these substitutions.

## II. DIVERSITY WITHIN THE ENDOMEMBRANE SYSTEM

The endomembrane system encompasses the majority of eukaryotic organelles, including transport systems for protein targeting, flagellum construction, nuclear transport and other features. Proteins forming coats, tethers, specificity modules (i.e. providing address codes for identification of specific compartments) and factors mediating formation and stability of these organelles almost exclusively belong to large paralogous families, with many of the organelle‐specific paralogues having already evolved prior to the LECA (reviewed in Dacks & Field, 2018). The most prominent and best‐supported proposal for how the organelles of the endomembrane system evolved (Dacks & Field, 2007) suggests that organelle‐specificity proteins, which participate in transport vesicle formation, compartment specificity and membrane fusion, generally expanded by gene duplication and co‐evolution of interacting proteins to allow organelle differentiation, with new compartments generated iteratively (Fig. 2 top panel). A special example of this recognises the architecture and homology between α‐solenoid/β‐propeller fold‐containing proteins that form vesicle coats, nuclear pore complexes (NPCs) and intraflagellar transport (IFT) complexes as evidence for common descent (Rout & Field, 2017). Comparative genomics established that a fully fledged organelle and protein complement was already present in the LECA (Dacks & Field, 2018), highlighting that gene expansion is not the sole process shaping endomembrane systems, and that loss clearly contributes towards specialisation. These paralogous families exhibit conservation ranging from ubiquitous and/or near universal to infrequently retained yet present in a wide range of taxa, indicating a presence in the LECA and underscoring the high frequency of secondary loss.

One comprehensively studied family is heterotetrameric adaptor complex‐containing coats. The best‐known examples are those containing adaptins and membrane‐deformation proteins such as clathrin, but the family also includes coat protein complex I (COPI) and TSET (Dacks & Robinson, 2017). While COPI mediates transport within the Golgi complex, the remaining complexes function in post‐Golgi transport and/or endocytic pathways (Dacks & Robinson, 2017). The distribution of this family illustrates the full span of evolutionary patterns. The adaptor protein 1 (AP‐1) complex and the associated clathrin coat, as well as COPI, appear essentially universal. AP‐2, involved in endocytosis from the cell surface, is also very well retained, with the striking exception being loss in African trypanosomes (Manna, Kelly & Field, 2013). The intestinal parasite *Giardia lamblia*, despite being highly reduced, retains AP‐1, AP‐2, and COPI (Touz *et al*., 2012), but other complexes seem more dispensable. The AP‐3 complex, which operates between the trans‐Golgi network to, from and between various types of endosomes (Simpson *et al*., 1997; Theos *et al*., 2005), has been lost at least four times, and the AP‐4 complex lost multiple times (Dacks & Robinson, 2017). The AP‐5 complex has been lost even more frequently (Ebenezer *et al*., 2019). Finally, TSET, which, similarly to AP‐2, may function in an endocytic pathway from the cell surface, is present in diverse eukaryotes (Hirst *et al*., 2014). However, it also has been lost frequently (Lee *et al*., 2015; Richardson & Dacks, 2022), and, strikingly, is absent from animal and fungal model systems (Hirst *et al*., 2014). This particular taxonomic distribution of being widespread in eukaryotes but not in well‐studied model systems is increasingly apparent and has relevance to creating truly generalised cell biological models (More *et al*., 2020). There are also cases where model organisms possess expanded AP complex complements, best characterised in metazoans where tissue‐ or cargo‐specific subunit expression occurs (Boehm & Bonifacino, 2001). However, expanded complements are present in *Trichomonas vaginalis* (Carlton *et al*., 2007) and embryophyte plants, and appear to be the result of recent convergent duplications (Larson, Dacks & Barlow, 2019). Moreover, there are notable instances of new cargo adaptors working as monomers such as the Golgi‐localised γ ear‐containing Arf‐binding (GGA) proteins (Hirst, Lindsay & Robinson, 2001), which are homologous to the adaptin γ interaction domain and can represent functions of single subunits or partial losses. For the TSET complex, the remaining medium subunit serves as a novel clathrin adaptor (Umasankar *et al*., 2014; Zaccai *et al*., 2022). Overall, the observed pattern of strict conservation of some complexes and loss of others is interpreted as sculpting (Elias *et al*., 2012), i.e. cutting away proteins and pathways during cellular system evolution. However, sculpting is not the sole process involved in the evolutionary dynamics of the endomembrane system.

Backfilling, the occurrence of equivalent function being undertaken by non‐homologous components, can be seen in the machinery mediating interactions between conserved components. Of several reported modes, the major endocytic pathway is supported by the protocoatomer family member clathrin, which is also involved in additional transport and sorting pathways (Robinson, 2015). The clathrin heavy chain is retained in the vast majority of eukaryotes, where it performs a well‐observed role, serving as one of the several interaction hubs operating within the endosomal system (Schmid & McMahon, 2007). By contrast, the regulatory light chain is less frequently detected and at least in the diplomonad flagellates (including *G. lamblia*), appears to have been functionally replaced by an analogous protein (Santos *et al*., 2022). Furthermore, only few of the conventional clathrin protein partners, i.e. those identified in animals and fungi, are present in trypanosomatids, and comparisons across eukaryotic lineages reveal that many are indeed specific to animals and fungi, or even just to metazoans (Field, Gabernet‐Castello & Dacks, 2007). An exception is the retention by trypanosomatids of epsin N‐terminal homology (ENTH) and AP180 N‐terminal homology (ANTH) domain containing proteins, which possess a phosphoinositide‐binding domain at the N‐terminus and clathrin‐interaction boxes within a largely disordered C‐terminal domain (Gabernet‐Castello, Dacks & Field, 2009; Manna *et al*., 2015). However, trypanosomatids do possess a significant cohort of distinct clathrin interactors, many of which have been identified directly from affinity isolation studies and validated in both African and American trypanosomes (Adung'a, Gadelha & Field, 2013; Manna *et al*., 2015; Kalb *et al*., 2016). A minority function in association with clathrin and share architectural similarities with the ANTH‐domain proteins (Manna *et al*., 2015), but without obvious sequence homology. The majority, however, are clearly even more highly divergent and not shared beyond these flagellates (Adung'a *et al*., 2013; Manna *et al*., 2015). This suggests that the trypanosome lineage has most likely lost many canonical genes but that this reduced repertoire has been ‘backfilled’ by the recruitment/emergence of new factors.

Further examples of backfilling have been identified in the trypanosome endocytic system, particularly in case of Ras‐related in brain (Rab) proteins. Rabs are identity markers coordinating vesicle fusion through interactions with tethering complexes, and facilitate transport through cytoskeletal elements (Stenmark, 2009; Kjos *et al*., 2018). Rab11 controls a major recycling pathway returning molecules to the cell surface after internalisation, a role conserved in trypanosomatids (Jeffries, Morgan & Field, 2001; Umaer, Bush & Bangs, 2018). In metazoans, Rab11 functions are mediated in part *via* coiled‐coil Rab11‐family interacting proteins (FIPs) and additional interactions with Sec15, a subunit of the exocyst, a heterooctameric complex responsible for late stages of exocytosis (Synek, Sekereš & Zárský, 2014). While FIPs are Metazoa‐specific, the Sec15–Rab11 interaction is maintained in *T. brucei*, but with two novel proteins supporting the interactions likely similar to those provided by FIPs. One, Rab11‐binding protein of 74 kDa (RBP74), interacts with both Rab11 and Rab5, shares a coiled‐coil architecture with FIPs and maintains a role in coordinating endocytosis and recycling, despite lacking sequence similarity to FIPs. The second is Exo99, a novel ninth subunit of 99 kDa within the trypanosomatid exocyst. The eight canonical exocyst subunits share similar overall architecture (Mei *et al*., 2018), suggesting an ancestral complex possessing one or few subunits. While there are many examples of canonical exocyst subunit expansion or loss (Žárský *et al*., 2020), Exo99 has no similarity to the canonical subunits, and is predicted to be architecturally related to α‐solenoid/β‐propeller fold‐containing proteins. Exo99 is essential for complex functionality (Boehm *et al*., 2017), and present throughout the trypanosomatids (Boehm & Field, 2019) suggesting recruitment of a novel protein to build a new version of the exocyst.

The final process under consideration is churning, specifically the birth and death of proteins of the same paralogous family, rather than replacement by non‐paralogous proteins. Good examples are once again Rab GTPases. The Rab complement of extant eukaryotes ranges widely, from only five in *Neopyropia* (formerly *Pyropia*) *yezoensis*, to 12 in yeasts, over 60 in Metazoa and several hundred in *Entamoeba* and *Trichomonas* (Diekmann *et al*., 2011; Elias *et al*., 2012; Klöpper *et al*., 2012; Petrželková & Eliáš, 2014). Reconstructions of the LECA identify between 19 and 24 ancient Rab subfamilies, depending on how subfamilies are categorised (Diekmann *et al*., 2011; Elias *et al*., 2012; Klöpper *et al*., 2012). Clearly loss sculpted this protein family, but examples of churning can be discerned. The first is metazoan Rab32, which plays roles within the endocytic system, but also in several endoplasmic reticulum (ER) functions (Wasmeier *et al*., 2006; Ortiz‐Sandoval *et al*., 2014). Rab32A and Rab32B are ancient Rab32 paralogues, with Rab32B lost from plants, fungi and the base of the vertebrate lineage (Elias *et al*., 2012; Ortiz‐Sandoval *et al*., 2014). However, Rab32A has been duplicated at least twice, to give rise to Rab29 (at the base of the holozoan radiation, i.e. the group encompassing animals and their single‐celled relatives) and Rab38 in vertebrates. Rab29 and Rab38 retain similar functions with Rab32, and provide clear examples of loss followed by re‐expansion within a protein family (Wasmeier *et al*., 2006; Ortiz‐Sandoval *et al*., 2014; Wang *et al*., 2014). More striking examples of changes to Rab complement come from land plants. Here, ancestral losses in the Archaeplastida supergroup removed over seven ancestral Rab subfamilies, with further loss of another five later in evolution in the lineage leading to land plants (Petrželková & Eliáš, 2014). As a consequence, the ancestral multi‐cellular plant, i.e. embryophyte, possessed a reduced Rab complement, specifically lacking Rabs associated with flagellar function (Rab28, Rabl2, IFT27) and endocytosis/phagocytosis (Rab4, 20, 22, 32A/B, 34) (Rutherford & Moore, 2002; Petrželková & Eliáš, 2014). Reduction in the endocytic Rab complement has been compensated by backfilling from Rab5 and Rab11, which act at early and recycling endosomes, respectively (Stenmark, 2009), as there is evidence for the emergence of a new Rab5 paralogue (RabF1/ARA6) in the Chloroplastida stem lineage, and expansion of Rab11 paralogues in Embryophyta (Petrželková & Eliáš, 2014).

An example that is intermediate between backfilling and churning is provided by the class C core vacuole/endosome tethering (CORVET) and homotypic fusion and vacuole protein sorting (HOPS) complexes within ciliates. This multi‐subunit tethering assembly functions in the process of vesicle fusion, interacting with Rab5 and Rab7 during early endosome maturation into late endosomes. These complexes are formed of four core subunits [vacuolar protein sorting‐associated protein (Vps) 11, 16, 18, and 33], with CORVET‐(Vps3 and 8) or HOPS‐(Vps39 and 41) specific subunits (Balderhaar & Ungermann, 2013; Solinger & Spang, 2013). The CORVET/HOPS complexes are an ancient eukaryotic feature (Field *et al*., 2007; Klinger, Klute & Dacks, 2013), but their specific subunits are missing from some key lineages, including apicomplexans and ciliates (Woo *et al*., 2015). Vps8 subunits expanded at the base of the oligohymenophorean ciliate class or potentially earlier, and HOPS subunits were subsequently lost, with the Vps8 paralogues replacing HOPS subunit function (Sparvoli *et al*., 2018, 2020). As the Vps8 subunit is distantly related to the lost Vps39 and Vps41 subunits, this represents churning, but since it is not an expansion of the same paralogue, it is also an example of backfilling. Additional losses of multi‐subunit tethering complexes in ciliates have also been suggested, raising the possibility of additional non‐homologous subunit replacement (Richardson & Dacks, 2022).

## III. REPLACEMENTS AND LOSSES IN THE NUCLEUS

The evolutionary processes described above have also contributed to shaping other organelles, including the nucleus. Despite conserved and central roles of the organelle, nuclear composition varies considerably across eukaryotes. Although the morphology of the nuclear envelope (NE) is remarkably conserved, the origins of many protein components indicate compositional diversity. The NE is punctuated by NPCs and also other structures such as the mitotic spindle and spindle pole body (SPB). In many lineages a proteinaceous lamina is present on the NE inner face (Gruenbaum *et al*., 2003). Mitotic division is supported by a tubulin spindle in most eukaryotes. We highlight several examples of protein complexes to illustrate the range of divergence for nuclear structures.

Nuclear division occurs *via* a multitude of distinct mechanisms. Association of specific microtubule organising centres (MTOCs), such as the SPB or centrosomes, with anchoring of the spindle at the NE is neither universal, mechanistically conserved, nor even always the same in different developmental stages within the same organism (Devos, Gräf & Field, 2014; Bouhlel *et al*., 2015; Ito & Bettencourt‐Dias, 2018). Spindle‐organising MTOCs are structurally conserved, with a 9 + 0 tubulin architecture in common with the 9 + 2 arrangement of axonemes, attesting to shared origin. However, SPBs in fungi and centrosomes in animal cells also exhibit clear divergence, offering a strong example of sculpting. Beyond the tubulin core, both SPBs and centrioles contain an additional subcomplex, the pericentriolar matrix (PCM). Multiple proteins are shared between the SPB and the centrosome PCM, but several subunits are unique to centrosomes or SPBs. Elegant reconstruction of PCM evolution suggests a pattern of secondary losses from a more complete ancestral form (Ito & Bettencourt‐Dias, 2018) and is supported by the presence of centrosome‐specific components in the PCM of basal fungi. Hence, some SPB PCM subunits have been acquired post‐loss of centrosome‐specific proteins during fungal evolution.

Eukaryotic genomes are packaged as chromatin, but remarkably, even this fundamental aspect of nuclear biology exhibits diversity. In most lineages, including animals, plants, fungi and almost all protists, histones comprise the chromatin protein core. Five core histones are recognised, with H2A, H2B, H3, and H4 forming the nucleosome and the multifunctional ‘linker’ histone H1 completing assembly and stabilising higher‐order chromatin structures. Several divergent histones are also present and employed to mark a DNA strand for specific functions, including initiation/termination of transcription and binding of kinetochores during mitosis. These major isoforms are ancient, present in the LECA and, together with some of the variant histones, quite probably universal (Stevens *et al*., 2020; Ding *et al*., 2021; Osakabe & Molaro, 2023), *albeit* with the likelihood of some churning taking place such that some histone variants have been generated post‐LECA and paralogues of some LECA histones lost. In the absence of detailed reconstruction of histone evolution, however, the mechanism and modes of diversification of this central eukaryotic gene family remain to be fully addressed.

In dinoflagellates (Alveolata), a significant alteration has occurred, exemplifying both replacement and extreme divergence. In this lineage histones are hyperdivergent, with extended N‐ and C‐termini, and are supported by additional core chromatin proteins (Gornik *et al*., 2019). Dinoflagellates have very high DNA content (>30‐fold that of Metazoa), which likely necessitates unusual packaging mechanisms. Core histones are at very low abundance and contain variant canonical residues compared to those of animals (Marinov & Lynch, 2016; Riaz & Sui, 2018). Mutation patterns of canonical histone modification sites suggest that dinoflagellate histones retain canonical functions in transcription (Gornik *et al*., 2019), but two additional protein families accompany dinoflagellate histones: histone‐like basic proteins (HLPs) and dinoflagellate/viral nucleoprotein (DVNP). Both likely originated from lateral gene transfer (LGT) events: the former from bacterial histone‐like proteins (Wong *et al*., 2003) and the latter from viruses (Gornik *et al*., 2012). Interestingly, HLPs are represented by two distinct subfamilies derived from different bacterial progenitors (Janouškovec *et al*., 2017*a*), leading to a model whereby DVNP proteins were acquired early, which facilitated increased genome compaction, with HLPs acquired later from at least two LGT events. While LGT is an unusual mechanism for supply of new proteins in eukaryotes, this is an excellent example of a case of such provision, most likely facilitating functional divergence of otherwise highly conserved proteins.

The nuclear lamina provides structural support and organisational functions to the nucleus. There are multiple molecular forms, but the system in animal cells likely represents the LECA state (Koreny & Field, 2016). In Metazoa and many other taxa, the lamina is comprised of lamins that assemble into higher‐order filaments. Viridiplantae and Euglenozoa lack detectable lamin orthologues, and lamina functions are performed by distinct protein systems, the nuclear matrix constituent proteins (NMCPs) in plants and nuclear pore proteins (NUPs) 1 and 2 in glycomonads (the subphylum encompassing kinetoplastids and diplonemids) (see online Supporting Information, Table S1; Koreny & Field, 2016; Groves *et al*., 2020; Butenko *et al*., 2021). NUP1 and 2 are repetitive, coiled‐coil proteins forming networks, with both the N‐ and C‐termini of NUP‐1 acting as interaction hubs, an assembly distinct from metazoan lamins (DuBois *et al*., 2012; Maishman *et al*., 2016; Padilla‐Meija *et al*., 2021). NUP1 and 2 position NPCs and silence subtelomeric genes while providing structural support, similar to the animal lamina (DuBois *et al*., 2012). However, since NUP1/2 are unrelated to lamins, this indicates a striking replacement of lamins during the emergence of glycomonads, or even earlier, as conventional lamins were not identified in *Euglena gracilis* (Koreny & Field, 2016). Similarly, the plant NMCP proteins also possess considerable coiled‐coil regions and multiple NMCP paralogues that expanded from an ancestral gene on several occasions (Ciska & Moreno Diaz de la Espina, 2013). Since metazoan‐related lamins are widespread amongst different eukaryotic lineages (Table S1), the most parsimonious model is backfilling, with loss followed by replacement, although the alternative, where NUP1 and 2 or NMCP evolved alongside lamins before their loss is also possible.

Monocentric kinetochores recognise centromeric chromosomal regions, linking chromosomes to the spindle during cell division. Inner and outer kinetochore subcomplexes are recognised, the inner interacting with centromeric DNA, usually *via* recognition of a variant histone H3, with the outer complex recruiting spindle microtubules. In nearly all taxa, kinetochores comprise many distinct proteins (Table S2), probably arising by duplication of genes in a stepwise manner (Musacchio & Desai, 2017; Field, 2019; Tromer *et al*., 2019). The trypanosomatid kinetochore stands as the sole known exception (Akiyoshi & Gull, 2014). While also complex and apparently functioning analogously to canonical kinetochores, there is only weak evidence for conservation of any component (Table S2; Akiyoshi & Gull, 2014; D'Archivio & Wickstead, 2017; Ebenezer *et al*., 2019; Butenko *et al*., 2020). The trypanosome lamina and kinetochore proteins interact and probably function together during chromosomal segregation (D'Archivio & Wickstead, 2017; Padilla‐Meija *et al*., 2021). Recent evidence of homology between some trypanosomatid kinetochore proteins and components of the eukaryotic synaptonemal complex suggests that trypanosomatid kinetochores may have evolved by repurposing meiotic components already present in the LECA, and supporting a fully unique origin (Tromer *et al*., 2021). Again, the most parsimonious model is backfilling, with loss followed by replacement, especially as potential ancestral kinetoplastid kinetochore components were already present in the LECA, although alternative pathways cannot be excluded.

The NPC is responsible for transport across the NE and many additional functions. It is comprised of several subcomplexes that together ensure selective transfer of cargo molecules. Morphologically the NPC is highly conserved, exhibiting eight‐fold symmetry and comprising concentric proteinaceous rings surrounding a central transport channel. Fibrous extensions are present at both nuclear and cytoplasmic NPC faces, with roles in regulating transport and messenger RNA (mRNA) maturation. Although much of the transport mechanism and core NPC structure is conserved across eukaryotes (Field & Rout, 2019), salient differences are present. Many of these are only apparent using high‐resolution morphological analysis (Makarov, Padilla‐Mejia & Field, 2021). There is one example, however, where detailed information is available for NPC remodelling. In trypanosomes, the NPC has lost components of the RNA quality control system, which actively monitor the integrity and functionality of RNA molecules and are normally located at the NPC cytoplasmic face (Obado, Field & Rout, 2017). This surveillance system ensures that defective mRNAs are not translated and, hence, that *cis*‐splicing is correct. As trypanosomal splicing is restricted to 5′ untranslated region (UTR) *trans*‐splicing, with *cis‐*splicing almost absent, this verification step seems to have become superfluous. Retention of this NPC subcomplex in euglenids and diplonemids, both of which do exhibit extensive *cis*‐splicing, supports this interpretation of a clear example of secondary loss in trypanosomatids (Butenko *et al*., 2021). Additional changes in the trypanosome NPC are associated with losses and possible replacements, including proteins of the nuclear basket, where the canonical subunits are replaced by a trypanosome‐specific analogue (Holden *et al*., 2014). Overall, we suggest that the NPC represents an example where multiple evolutionary mechanisms likely contributed, but that loss of subunits is clear and this may well have led to additional innovation. In these examples, the NPC has most probably been backfilled with the acquisition of novel components.

## IV. EXTREME DIVERGENCE OF MITOCHONDRIAL PROTEIN COMPLEXES

Mitochondria, the most prominent organelle of endosymbiotic origin (Martin & Mentel, 2010), contribute many essential functions. They are highly specialised in morphology and composition and show extensive diversity (Pánek *et al*., 2020). In trypanosomes and related flagellates, mitochondria harbour a structurally unusual genome (Jensen & Englund, 2012), the transcripts of which undergo extensive editing and processing (Aphasizheva *et al*., 2020). Some anaerobic protists are completely devoid of mitochondrial DNA and instead of canonical mitochondria retain mitochondrion‐related organelles (MROs). These MROs are not involved in oxidative phosphorylation, but perform other functions (Leger *et al*., 2019); MROs have been excluded from our comparative analysis as they represent a special case of gene loss.

### (1) Mitochondrial import

In the course of endosymbiotic integration, most mitochondrial genes were either lost or relocated to the nuclear genome (Roger, Muñoz‐Gómez & Kamikawa, 2017). Consequently, nuclear‐encoded proteins are conveyed into the organelle *via* dedicated import systems (Wiedemann & Pfanner, 2017). There are at least five major pathways for protein import to mitochondria, which require distinct machineries, including TOM and TIM (translocase complex of the outer and inner membranes, respectively), SAM (sorting and assembly machinery), and MIM (mitochondrial import machinery) (Wiedemann & Pfanner, 2017). For the purposes of this review, we will focus on the two former complexes that mediate import of presequence‐containing (TOM and TIM23 complexes) and polytopic hydrophobic carrier (TOM and TIM22) proteins, respectively.

The TOM complex is composed of a pore‐forming, bacteria‐derived, Tom40, associated receptors (Tom20, 22 and 70) and various chaperones (Bausewein *et al*., 2020; Wang *et al*., 2020). As the central pore, Tom40 is retained in essentially all eukaryotes [Fig. S1 (see Fig. S0 for information on how to read the dartboard charts); Table S3]. The Tom22 receptor, playing an important role in oligomerisation of the TOM complex, is also nearly ubiquitous. However, Tom70 and Tom 20 appear to have been lost on multiple occasions, which presumably reflects a non‐essential role in pore formation (Fig. S1; Table S3). Sculpting of the TOM complex by the loss of receptors in some lineages might be at least partly explained by their overlapping substrate specificities and the ability to compensate reciprocally for each other's absence (Yamano *et al*., 2008; Backes *et al*., 2018). TOM complex sculpting also involves so‐called small Toms (Tom5–7), which do not directly contribute to the protein translocation function (Wiedemann & Pfanner, 2017), with Tom5 and 6 likely present in the LECA but retained only in opisthokonts, and chloroplastids, while Tom6 is also preserved in some discobans (Fig. S1; Table S3). A clear case of a subunit replacement by its functional analogue is that of the ancestral Tom70‐like receptor by ATOM69 (atypical outer membrane translocase 69) and OM64 (outer mitochondrial membrane protein of 64 kDa) in trypanosomes and plants, respectively (Schneider, 2020). The trypanosomatid complex shares only Tom40/ATOM40 and Tom22/ATOM14 with other eukaryotic lineages, albeit most of the additional subunits are recognised as functional analogues of the canonical TOM subunits (Fig. S1; Table S3), a further example of recoating a conserved core with a divergent shell of interacting proteins (Fig. 1; Mani, Meisinger & Schneider, 2016).

Upon interaction with the TOM complex, presequence‐containing proteins destined for the mitochondrial matrix and metabolite carriers of the inner membrane (IM) are directed to their locations by the TIM23 complex and the TIM22 carrier‐insertase, respectively (Wiedemann & Pfanner, 2017). The core components of both complexes (pore‐forming Tim22 and Tim23; Tim17 closely associated with Tim23) are present in all eukaryotes possessing aerobic mitochondria. However, trypanosomes have retained only a single TIM17/22/23 family transporter (TbTim17), most closely related to TIM22 (Fig. S2; Table S4; Žárský & Doležal, 2016). Unique absence of Tim23 and a canonical eukaryotic Tim17 in trypanosomatids is, most likely, another example of a derived ‘simplification’, involving (secondary) gene loss. The most plausible scenario is that an initially neutral recruitment of *T. brucei* specific presequence translocase‐associated motor subunit 27 (TbPam27) and two rhomboid proteins (TimRhom I and II) gave TbTim17 the ability to translocate presequence‐containing substrates, thus making the function of the ancestral TIM23 complex redundant (Schneider, 2018). The losses of core subunits are only described in trypanosomatids and a few other groups, but mainly MROs in those latter instances (Pyrihová *et al*., 2018). However, losses of chaperones Tim8–10 and 13 are more common (Fig. S2; Table S4), and especially evident in the case of Tim8 and 13, forming a subcomplex, at least in yeast, and responsible for relocation of only a narrow group of precursor proteins. This may explain rather frequent loss of one or both paralogues (Fig. S2; Table S4).

In addition to highlighting gene losses, variations in TIM complex composition illustrate the existence of moonlighting proteins. Examples include the Sdh3 subunit of succinate dehydrogenase in *S. cerevisiae* (Gebert *et al*., 2011), NADH dehydrogenase (respiratory complex I) subunits B14.7‐like and Tim 23‐2 in *A. thaliana* (Wang *et al*., 2012), acyl glycerol kinase in Metazoa (Valpadashi *et al*., 2021), and acyl‐CoA dehydrogenase (ACAD) in *T. brucei*, which in addition to their more widely known functions are components of TIM translocase in these organisms (Singha *et al*., 2012). However, it is unclear whether the recruitment of these proteins to the TIM complex in certain lineages created functional redundancies, possibly allowing subsequent losses of some of the original subunits, or whether these proteins substituted for some ancestral subunits, already lost, instead.

### (2) Mitochondrial contact site and cristae organising system (MICOS)

As the name implies, the primary function of the MICOS is in organising cristae and anchoring them to the mitochondrial IM (Wollweber, von der Malsburg & van der Laan, 2017). In addition, MICOS interacts with the Sam50 component of SAM and TOM complex components to create contact sites between the IM and OM. In opisthokonts, MICOS is composed of two dynamic subcomplexes, organised around broadly conserved core proteins Mic10 and Mic60, respectively (Zerbes *et al*., 2012; Barbot *et al*., 2015). Mic60 contains a mitofilin domain originating from ancestral bacteria (Muñoz‐Gómez *et al*., 2015), and together with Mic10 and Mic19 traces back to the LECA (Fig. S3; Table S5). Mic12 is also proposed to have been present in the LECA (Huynen *et al*., 2016).

The trypanosomatid MICOS is also bipartite, although unlike opisthokont MICOS, is divided into integral and peripheral subcomplexes (Eichenberger *et al*., 2019). Altogether, trypanosomes possess eight MICOS subunits, with most being lineage specific, indicating a near‐complete replacement, albeit with a shared core of Mic60 and Mic10 (Huynen *et al*., 2016; Kaurov *et al*., 2018). TbMic60 and its orthologue in *E. gracilis* lack the mitofilin domain (Kaurov *et al*., 2018; Hammond *et al*., 2020), and the absence of the domain is suggested to be compensated by mitofilin domain‐containing TbMic34, a core component of the peripheral subcomplex (Kaurov *et al*., 2018; Eichenberger *et al*., 2019). TbMic60 has a domain architecture resembling that of the N‐terminus of the canonical Mic60, including a mitochondrial presequence, *trans*‐membrane and coiled‐coil domain (Kaurov *et al*., 2018). It seems that in Euglenozoa the ancestral Mic60 gene split into two parts, one encoding the core Mic60 region (=TbMic60) and the other encoding the mitofilin domain (TbMic34) (Fig. S3).

The euglenozoan MICOS also possesses a number of lineage‐specific components, among which is a novel thioredoxin‐like subunit, Mic20 (Fig. S3; Table S5), which performs a still unassigned role in the stabilisation of intermembrane space proteins and which may well represent a novel subunit and function (Kaurov *et al*., 2018, 2022). Whether major deviations from opisthokont counterparts, in both composition and structure of MICOS, similar to those observed in trypanosomes, occur in other lineages, is not known. Furthermore, the basic mechanism for cristae formation that originated from the ancestral bacteria was complexified by duplications, including Mic19 and 25 in opisthokonts and Mic10‐1/2 in trypanosomes and was later sculpted by gene loss and backfilled with novel subunits.

### (3) Oxidative phosphorylation system

Oxidative phosphorylation (OxPhos) is mediated through respiratory complexes I–IV and ATP synthase, all located in the IM. A number of core subunits of alphaproteobacterial origin are conserved, surrounded by subunits of eukaryotic origin, only some of which were already present in the LECA (Timmis *et al*., 2004; Roger *et al*., 2017).

Complex I, NADH:ubiquinone oxidoreductase, is the major entry for electrons into the respiratory chain (Brandt, 2006). In yeast, some plants, and myzozoans, there is a complete loss of complex I combined with alternative electron entry points (De Vries & Marres, 1987; Gardner *et al*., 2002; Petersen *et al*., 2015). By contrast, opisthokonts, euglenozoans and *Tetrahymena thermophila* possess extremely large complexes of ~40–60 subunits (Huynen & Elurbe, 2022).

Eukaryotes share a highly conserved set of 17 alphaproteobacterial subunits, which encompasses all redox centres and hence the catalytic core of the complex (Yip *et al*., 2011). Additionally, the LECA had incorporated a large number of accessory subunits, almost tripling subunit composition during early eukaryotic evolution (Cardol, 2011). While the losses of core subunits in eukaryotes are extremely rare, some accessory subunits are lost more frequently, with the notable example of a γ‐carbonic anhydrase absent in opisthokonts (Gawryluk & Gray, 2010). Gains of lineage/species‐specific accessory subunits are also very common. The acquisition of additional subunits occurred from various sources (Yip *et al*., 2011; Elurbe & Huynen, 2016), and most are products of gene duplications and/or recruitment from other mitochondrial protein families, suggesting paralogue expansion (Szklarczyk & Huynen, 2009; Elurbe & Huynen, 2016).

In *T. brucei* at least seven accessory subunits are encoded by what seem to be duplications of genes encoding other accessory subunits, either species/lineage‐specific, or more universal (Table S6; Duarte & Tomás, 2014). In some lineages novel accessory subunits even seem to form new functional modules (e.g. an extra matrix ‘FAS domain’ in *T. brucei*) (Duarte & Tomás, 2014). It was suggested that in the euglenozoan *Paradiplonema* (formerly *Diplonema*) *papillatum*, novel accessory subunits might have replaced some of the absent accessory proteins which are otherwise nearly universally distributed (Valach *et al*., 2018). Overall, our comparative analysis again confirms that 43 subunits were already part of complex I in the LECA (Fig. S4; Table S6).

Succinate:ubiquinone oxidoreductase (complex II) in opisthokonts consists of four proteobacteria‐derived subunits, with succinate dehydrogenase (SDH) subunit A (SDHA) and SDHB forming a soluble catalytic domain anchored in the IM by the hydrophobic anchor composed of SDHC and SDHD (Moosavi *et al*., 2019). While the catalytic subunits are universal (Fig. S5; Table S7; Huang & Millar, 2013), SDHC and SDHD appear to have been lost several times, for example in alveolates (Fig. S5; Evers *et al*., 2021). It was assumed that the accessory lineage‐specific subunits may have replaced the ancestral membrane‐anchoring ones in some groups (Maclean *et al*., 2022). However, the most recent experimental data hint that the genomes of these organisms encode extremely divergent integral membrane subunits (Mühleip *et al*., 2023), and the observed absences might be attributed to the limitations of bioinformatic tools. The genes encoding the subunits of proteobacterial origin underwent duplication in some lineages (e.g. in chloroplastids, some amoebozoans and stramenopiles) (Table S7). In Euglenozoa the *sdhb* gene has been split into two separately transcribed and translated parts. The separate protein products correspond to the N and C termini of a typical SDHB protein (Morales *et al*., 2009; Perez *et al*., 2014). In discobans, chloroplastids and alveolates, whenever complex II was experimentally characterised, the classical opisthokont four‐subunit composition turned out to be augmented by a variable number of additional subunits (Fig. S5; Table S7; Morales *et al*., 2009; Huang *et al*., 2019*b*; Evers *et al*., 2021). Of these, at least the homologues of plant SDH5 and SDH6 are readily identifiable outside the lineage and were likely present in the LECA (Huang *et al*., 2019*b*; Gray *et al*., 2020). This would imply that the LECA possessed a more complex succinate:ubiquinone oxidoreductase than model opisthokont (yeast and mammalian) systems, and that the observed bacteria‐like composition of complex II in that group is the result of ancient sculpting events.

Complex III, ubiquinol:cytochrome *c* oxidoreductase, forms homodimers (Berry *et al*., 2000). The catalytic engine incorporating cytochromes *b* and *c1*, and Rieske Fe‐S protein is of proteobacterial origin and is conserved across eukaryotes (Fig. S6; Table S8; Smith, Fox & Winge, 2012). The accessory subunits (COR1 and 2, QCR7‐10) involved in the stabilisation of complex III and the formation of III + IV supercomplexes (Maclean *et al*., 2022), can also be traced back to the LECA, with QCR8 and 9 either lost or diverged beyond recognition in several lineages (Fig. S6; Table S8). It is noteworthy that complex III of apicomplexans, euglenozoans, and a chloroplastid (*Chlamydomonas reinhardtii*) also acquired a small, extra cohort of species‐ or lineage‐specific subunits (Fig. S6; Table S8; Perez *et al*., 2014; Salinas *et al*., 2014; Evers *et al*., 2021). Some of the lineage‐specific subunits, such as QCRTB1 and QCRTB2 1 in Euglenozoa (Perez *et al*., 2014), again are the result of duplicating a gene encoding one of the LECA‐derived complex III subunits (COR1/QCR1) in the ancestor of the group.

Complex IV, also known as cytochrome *c* oxidase, which couples oxygen reduction to proton translocation across the IM, is surprising with regard to the amount of variation in subunits when comparing different protists to the well‐studied yeast and mammalian models (Zíková *et al*., 2008; Maclean *et al*., 2022; Zhou *et al*., 2022). Indeed, euglenozoans, alveolates and plants, in addition to the universally conserved catalytic core (subunits COX1–3) of proteobacterial origin and several supernumerary subunits demonstrating a patchier distribution among eukaryotes, have acquired multiple lineage/species‐specific complex IV components (Fig. S7; Table S9). These subunits are predicted to be involved in the assembly and regulation of the complex, in supercomplex formation, and in maintenance of its structural integrity and/or unusual species‐specific secondary functions (Perez *et al*., 2014; Evers *et al*., 2021; Zhou *et al*., 2022). Along with the accretion of novel subunits and the apparent losses of others in certain groups, gene duplication played a profound role in the evolution of complex IV (e.g. COX7A and B in mammals, COX6C in *T. thermophila*), including the duplications of genes encoding subunits of other respiratory complexes [e.g. the COX4/COX5b subunit is a duplication product of the NADH:ubiquinone oxidoreductase subunit S6 (NDUFS6) complex I subunit gene] (Elurbe & Huynen, 2016). Overall, complex IV in many eukaryotes is often somewhat expanded compared to its opisthokont counterpart and was mainly shaped, along with a few cases of losses and/or extreme divergence, by gene duplication of ancestral subunits and accretion of dozens of species/lineage‐specific subunits.

Complex V, or F_O_F_1_‐ATP synthase consists of the F_O_ moiety embedded in the IM, and the soluble F_1_ moiety extending into the matrix (Walker, 2013). The F_1_ subcomplex incorporating the catalytic head subunits α and β, as well as the γ, δ, and ε subunits forming the central stalk, is nearly universally conserved across eukaryotes. The very rare absences are probably the result of either incomplete data or limitation of bioinformatic searches (Fig. S8; Table S10). The only known conspicuous addition to the F_1_ subcomplex occurred in Euglenozoa, where the newly acquired subunit p18 is associated with the F_1_ head. This Euglenozoa‐specific component does not appear to contribute directly to the catalytic function of the enzyme but is essential for its integrity (Gahura *et al*., 2018). All other eukaryotic F_1_ subunits are conserved in Euglenozoa, which makes the incorporation of this novel subunit rather challenging to explain unless a neutral process is assumed, ending with this extra, chaperonin‐like subunit, compensating for progressive loss of stabilising protein–protein interactions between the F_1_ subunits.

The composition of the F_O_ subcomplex, except for the conserved subunits a and c which form the H^+^ channel, is more variable with several losses being evident (Fig. S8; Table S10). These losses include peripheral stalk subunits b and d in *Naegleria gruberi*, ATP8 in *Capsaspora owczarzaki*, Rhodophyta, and *Emiliania huxleyi*, and subunit h in several lineages. The peripheral stalk (stator) subunits putatively involved in ATP synthase dimerization (f, e, g, i/j, and k) demonstrate an even more patchy distribution (Fig. S8; Table S10). Experimental evidence indicates the presence of numerous species/lineage‐specific F_O_ subunits in alveolates, euglenozoans, chlorophyceans and amoebozoans (Vázquez‐Acevedo *et al*., 2006; Balabaskaran Nina *et al*., 2010; Perez *et al*., 2014). Some of these subunits, however, were recently found to be divergent homologues of the (nearly) universal subunits (Sinha & Wideman, 2023). The F_O_ subcomplex has undergone multiple losses along with gains of species/lineage‐specific subunits, hinting that backfilling might be a dominant mode of ATP synthase evolution. However, the results of functional studies in line with this assumption are currently not available, and it is not feasible to draw such conclusions based on comparative genomics data alone. Another view suggests that most if not all species/lineage‐specific subunits are accretions that do *not* replace missing subunits. Overall, experimental and comparative genomics analyses performed by us and others (Sinha & Wideman, 2023) suggest that the LECA complex V possessed 17 subunits (identified by experimental studies in at least two eukaryotic supergroups), which represent functional homologues across eukaryotes. There is one additional protein (ATPTG9) which was likely encoded in the LECA genome but this might not represent a *bona fide* complex V subunit (Fig. S8; Table S10).

### (4) Mitochondrial secretion system

Recently, a surprising new addition to the set of mitochondrial protein complexes was found. Multiple core homologues of a bacterial secretion system (type 2; T2SS) used to secrete proteins across the outer membrane of certain Gram‐negative bacteria are detected in the genomes of heteroloboseans, jakobids, malawimonads and hemimastigotes, while being absent from model opisthokonts (Horváthová *et al*., 2021). Some of these proteins are present in mitochondria, and from their experimental behaviour infer the presence of a T2SS‐derived system in mitochondria (miT2SS). The phylogenetic evidence and distribution patterns of key T2SS subunits across eukaryotes strongly indicate that a miT2SS pathway was present in the LECA (Horváthová *et al*., 2021). Direct experimental evidence is lacking but phylogenetic profiling identifies over 20 protein families (seven containing family members linked by, for instance, sequence features or functional data), with indications of peroxisomes as secretion targets. This adds to the ever‐increasing list of peroxisome–mitochondria connections (Schrader & Yoon, 2007). More important for our present discussion is that the preliminary genomic patterns of retention, loss, replacement and addition of putative miT2SS subunits mirrors the patterns we found for the other eukaryotic complexes. Details can be found in Horváthová *et al*. (2021).

## V. THE DEEP: PATTERNS OF VARIATION ACROSS EUKARYOTES

**Fig. 3:**
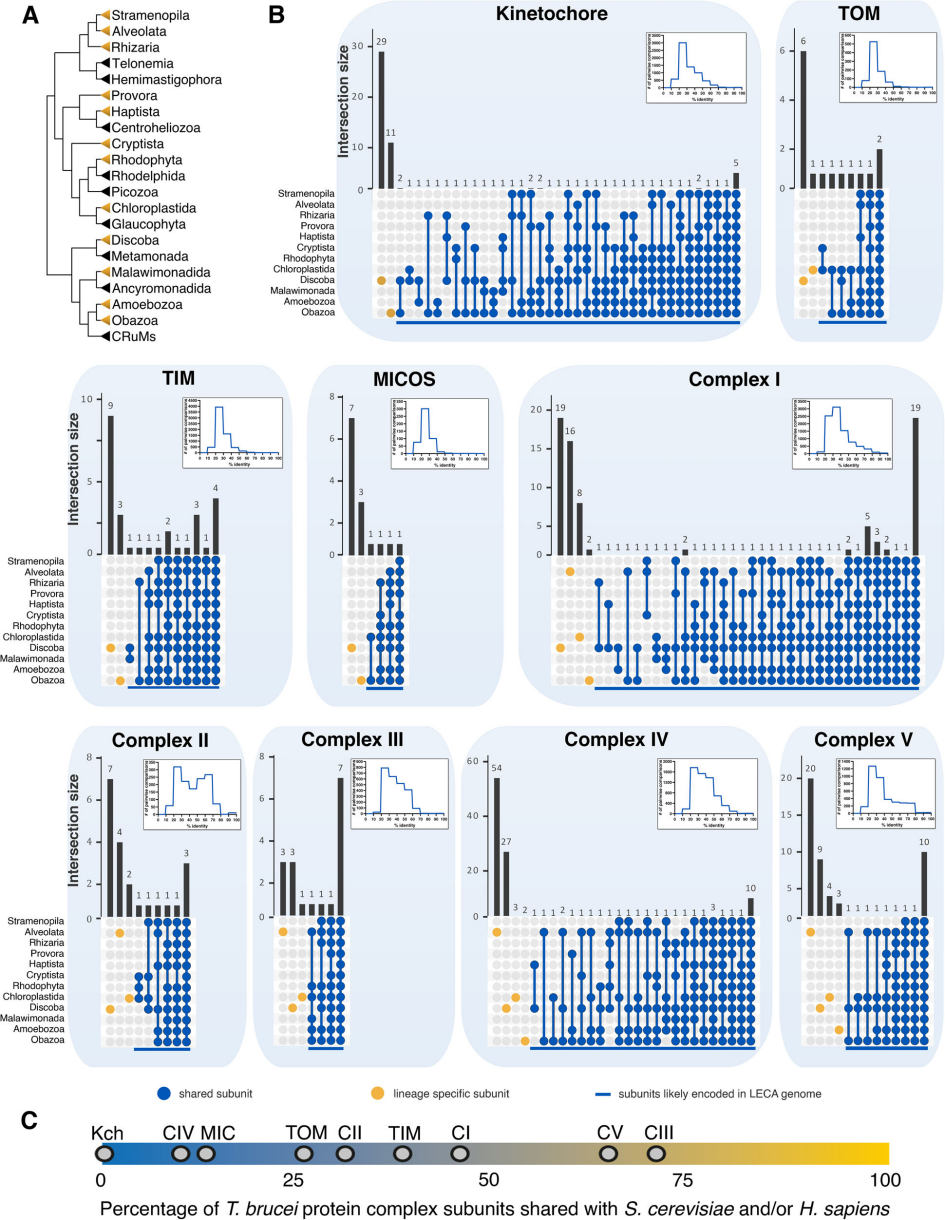
Relative conservation of selected protein complexes across eukaryotes. (A) A cladogram depicting the phylogenetic relationships among major eukaryotic supergroups. Taxa for which a comparative analysis was conducted in this study are marked in yellow. Based on Tice *et al*. (2021). CRuMs, clade consisting of Collodictyonidae, Rigifilida and *Mantamonas*. (B) Retention of individual subunits among eukaryotic supergroups is indicated by the number of lineage‐specific and shared subunits in UpSetR plots (Conway *et al*., 2017). A subunit was considered present in a supergroup when it was identified in at least one species from this group. Retention patterns for the subunits shared by two or more supergroups, and thus likely encoded in the genome of the last eukaryotic common ancestor (LECA), are shown in blue; lineage‐specific subunits are shown in yellow. See Tables S2–S10 for the species where a particular complex was experimentally characterised. Pairwise amino acid identities were calculated using the *EMBOSS* module in Biopython with the Needleman–Wunsch algorithm (Cock *et al*., 2009). Histograms depict distributions of pairwise per cent identities for shared subunits. (C) Distribution of *Trypanosoma brucei* (Euglenozoa) protein complexes with respect to the relative number of subunits shared with *Saccharomyces cerevisiae* and/or *Homo sapiens* (Opisthokonta). Positions of individual complexes on this scale are marked: Kch, kinetochore; MIC, mitochondrial contact site and cristae organising system (MICOS); CI–V, respiratory complex I–V. The total number of complex subunits in *T. brucei* is set at 100%.

Extensive *in silico* analysis of selected complexes was undertaken, specifically including taxonomically relevant organisms (Fig. 3A) with high‐quality genome assemblies to broaden understanding of complex evolution (Table S11; Fig. S9; Appendix S1) and revealed that the LECA genome encoded somewhere from four (for MICOS) to several dozen (for complexes I and IV) subunits for a respective protein complex (Fig. 3B). Moreover, for almost every lineage of extant eukaryotes for which experimental data exist, multiple species‐ or lineage‐specific subunits were identified (Tables S2–S10; Figs [Link], [Link]). The number of species/lineage‐specific subunits is comparable to, or even exceeds, the number of ancestral subunits predicted in the LECA, including the MICOS complex in *T. brucei* and respiratory complexes I and IV in alveolates and euglenozoans.

In some cases, species/lineage‐specific proteins form a completely novel functionally analogous complex replacing the ancestral eukaryotic machinery, with the kinetoplastid kinetochore (Fig. 3C) and plant and kinetoplastid lamins being prominent examples, whilst elsewhere species/lineage‐specific subunits form a divergent shell around conserved cores of ancestral subunits, for example, in TOM and most respiratory complexes. Functions of these supernumerary subunits include assembly and stabilisation, replacing the functions of lost ancestral subunits and facilitating the formation of supercomplexes (Huynen & Elurbe, 2022; Maclean *et al*., 2022).

Although complexification is a prevalent trend for the experimentally characterised set of complexes discussed herein, secondary simplification as the result of the loss of ancestral subunits is also observed (e.g. the loss of presumably ancestral γ‐carbonic anhydrase, and SDH5 and SDH6 subunits in case of the respiratory complexes I and II respectively in opisthokonts). Such divergent and relatively well‐studied organisms as *T. brucei* are valuable indicators of complex diversity, as experimental data are only very rarely available for species outside model opisthokonts and plants, which collectively represent only two eukaryotic supergroups. Combining experimental and *in silico* data (Fig. 3C) indicates that *T. brucei* shares somewhere between zero and 70% of complex subunits with opisthokonts. Extreme divergence of protein complexes is manifested not only in highly variable subunit composition, but also in low sequence similarity among the respective subunits (Fig. 3B).

## VI. DISCUSSION

Multiple reconstructions demonstrate that the LECA was a highly complex organism and in possession of a larger intracellular compartment cohort than many extant organisms (Koumandou *et al*., 2013; O'Malley *et al*., 2016; Wideman & Muñoz‐Gómez, 2016). Such a high level of complexity is further demonstrated by the combined experimental and *in silico* evidence discussed here. Many modes for evolution of gene families and protein complexes have been proposed, including duplication, churning, backfilling and sculpting (Fig. 1; Elias *et al*., 2012). While sculpting is simply carving away of genes encoding protein subunits, churning is exemplified by rapid birth and death of new paralogues. Backfilling is a two‐step process by which a subunit gene is lost, but retention of functional integrity, and even functional diversification, is facilitated through expansion of other genes within the pathway or complex (Gray *et al*., 2010; Padilla‐Meija *et al*., 2021). We initially defined backfilling by considering paralogue expansion only, as exemplified by the HOPS/CORVET and Rab32 examples here, but we found broader cases, where non‐paralogous genes can act, as in the case of TOM and MICOS complexes. Further, each mechanism can operate in combination, such that sculpting followed by backfilling is in essence a special example of churning. While all of these events seem widely attested to shape evolution of eukaryotic protein complexes, we suggest that gene loss is a major initiator, as loss can generate space for a new gene (Dacks & Field, 2018). Importantly, the processes described provide an opportunity for selection to operate, but the initial loss itself should be considered as essentially neutral.

We provide examples in support of these multiple scenarios from simple loss to replacement of entire structures by apparently unrelated proteins. The presence of large paralogous families populating much of the endomembrane system provides a flexible means by which cells can evolve, as new paralogues can be accommodated within existing machinery. However, even within the less closely related gene cohorts of mitochondrial complexes, the presence of multiple paralogues is noteworthy. Furthermore, the ability of these paralogous families to recruit and adapt a shell of less‐retained proteins to augment function is broadly similar for many of the complexes considered here. *In silico* evidence highlights the patchy retention of many subunits surrounding a conserved core, emphasising the frequency and hence importance of such events.

Overall, this suggests a potent role for CNE. While gene loss is in itself a comparatively minor evolutionary event and facilitated by an environment where the lost gene product presumably has little or no impact on fitness, additional factors including population size and the stability or dynamic nature of the environment are of importance for fixing a new allele, or the irreversible loss of a gene. We suggest that this combination of factors provides a mechanism for the evolution of novelty. Thus, paradoxically, gene loss is a major evolutionary force driving divergence of the highly elaborate eukaryotic protein complexes.

## VII. CONCLUSIONS

(1) We present multiple scenarios for evolution of protein complexes, with most incorporating a gene loss event.
(2) In many complexes a core of subunits coordinates an interacting shell of lineage‐specific components.
(3) There are examples of entire complexes, considered to be indispensable, being replaced by functional analogues.
(4) We propose that gene loss is a major evolutionary force for divergence by providing opportunity for novel subunit recruitment.

## Supporting information


**Appendix S1.** Supplementary materials and methods.

**Fig. S0.** How to read the dartboard charts.

**Fig. S1.** Dartboard chart representation of the translocase of the outer mitochondrial membrane (TOM) complex.

**Fig. S2.** Dartboard chart representation of the translocase of the inner mitochondrial membrane (TIM) complex.

**Fig. S3.** Dartboard chart representation of the mitochondrial contact site and cristae organising system (MICOS) complex.

**Fig. S4.** Dartboard chart representation of respiratory chain complex I (NADH:ubiquinone oxidoreductase).

**Fig. S5.** Dartboard chart representation of respiratory chain complex II (succinate: ubiquinone oxidoreductase).

**Fig. S6.** Dartboard chart representation of respiratory chain complex III (ubiquinol:cytochrome *c* oxidoreductase).

**Fig. S7.** Dartboard chart representation of respiratory chain complex IV (cytochrome *c* oxidase).

**Fig. S8.** Dartboard chart representation of complex V (F_O_F_1_‐ATP synthase).

**Fig. S9.** Genome/transcriptome completeness for the species in the reference data set estimated using genome/transcriptome‐derived proteins as the query and eukaryota_odb10 as the database in BUSCO v5.4.3 (Manni *et al*., 2021).

**Table S1.** Constituents of the lamina complex and their amino acid sequences across eukaryotes.
**Table S2.** Constituents of the kinetochore complex and their amino acid sequences across eukaryotes.
**Table S3.** Constituents of the translocase of the outer mitochondrial membrane (TOM) complex and their amino acid sequences across eukaryotes.
**Table S4.** Constituents of the translocase of the inner mitochondrial membrane (TIM) complex and their amino acid sequences across eukaryotes.
**Table S5.** Constituents of the mitochondrial contact site and cristae organising system (MICOS) complex and their amino acid sequences across eukaryotes.
**Table S6.** Constituents of respiratory complex I (NADH:ubiquinone oxidoreductase) and their amino acid sequences across eukaryotes.
**Table S7.** Constituents of respiratory complex II (succinate:ubiquinone oxidoreductase) and their amino acid sequences across eukaryotes.
**Table S8.** Constituents of respiratory complex III (ubiquinol:cytochrome *c* oxidoreductase) and their amino acid sequences across eukaryotes.
**Table S9.** Constituents of respiratory complex IV (cytochrome *c* oxidase) and their amino acid sequences across eukaryotes.
**Table S10.** Constituents of respiratory complex V (F_O_F_1_‐ATP synthase) and their amino acid sequences across eukaryotes.
**Table S11.** Reference data set used in our study.
