# Supplementary material for: Lessons from the deep: mechanisms behind diversification of eukaryotic protein complexes
Source: Biol Rev Camb Philos Soc. 2023 Jun 19;98(6):1910–27. doi: 10.1111/brv.12988 (PMC10952624; doi:10.1111/brv.12988)
Supplement: Supplementary file 1 — Appendix S1. Supplementary materials and methods. [file BRV-98-1910-s002.docx]

**Appendix S1. Supplementary materials and methods**

Nuclear and mitochondrial genome/transcriptome assemblies for 23 species representing eukaryotic diversity were downloaded from the sources specified in Table S11 and used as a reference data set for comparative genomics analyses*.* The completeness of the reference genome and transcriptome sequences was estimated using BUSCO v5.4.3 in protein mode and eukaryota_odb10 as the database (Manni *et al*., 2021). The transcriptome assembly of *A. twista* showed signs of contamination with its bodonid prey (Janouškovec *et al*., 2017*b*) and bacteria, and therefore we excluded putative protein complex components giving top BLAST hits to kinetoplastid and bacterial sequences from the final results. For identification of complex subunits, accessions for proteins identified experimentally and bioinformatically were collected from the relevant literature sources specified in Tables S1–S10 and used as initial BLASTp (Camacho *et al*., 2009) queries (E-value threshold 1*e*-5) for searching the sequences of interest in the reference genome data set. The validity of BLASTp hits was confirmed using a reciprocal BLAST approach. Retrieved protein sequences were used for building initial Hidden Markov models (HMMs) which, along with the individual sequences, were used as queries in the AMOEBAE pipeline with the default settings (https://github.com/laelbarlow/amoebae). An updated set of HMMs was built based on the results of the AMOEBAE pipeline and used for the second round of searches using HHMER v. 3.3.2 (http://hmmer.org/) with the default settings. When validation of the hits by reciprocal BLAST was not possible due to high sequence divergence, we used HHpred with Pfam-A v.35 and PDB70 databases to validate homology. For creating HMMs for putatively lineage-specific proteins, each query sequence was BLASTed against the NCBI non-redundant protein database with an *e*-value threshold of 1*e*-20, minimum query coverage and sequence per cent identity set to 60% and 40%, respectively. To screen for weak similarity between putative lineage-specific and more widespread protein complex subunits, we employed more sensitive profile-to-profile comparisons (HHpred) implemented in HHSuite v.3.3.0 (Steinegger *et al.*, 2019) with the default settings using Pfam-A v.35, PDB70 and a custom set of protein complex subunits as databases. For the identification of lamins, the Pfam database entry for lamin tail domain (PF00932) was used as a query along with the custom HMM, and the hits were inspected for the presence of a typical lamin domain architecture (Kollmar, 2015). Centromere histone H3 candidates were selected based on several specific sequence features as described previously (Butenko *et al*., 2020).

**References**

Butenko, A., Opperdoes, F. R., Flegontova, O., Horák, A., Hampl, V., Keeling, P., Gawryluk, R. M. R., Tikhonenkov, D., Flegontov, P. & Lukeš, J. (2020). Evolution of metabolic capabilities and molecular features of diplonemids, kinetoplastids, and euglenids. *BMC* *Biology* **18**, 23.

Camacho, C., Coulouris, G., Avagyan, V., Ma, N., Papadopoulos, J., Bealer, K. & Madden, T. L. (2009). BLAST+: architecture and applications. *BMC Bioinformatics* **10**, 421.

<https://github.com/laelbarlow/amoebae>

http://hmmer.org/

Janouškovec, J., Tikhonenkov, D. V., Burki, F., Howe, A. T., Rohwer, F. L., Mylnikov, A. P. & Keeling, P. J. (2017*b*). A new lineage of eukaryotes illuminates early mitochondrial genome reduction. *Current Biology* **27**, 3717–3724.e5.

Kollmar, M. (2015). Polyphyly of nuclear lamin genes indicates an early eukaryotic origin of the metazoan-type intermediate filament proteins. *Scientific Reports* **5**, 10652.

Manni, M., Berkeley, M. R., Seppey, M., Simão, F. A. & Zdobnov, E. M. (2021). BUSCO update: novel and streamlined workflows along with broader and deeper phylogenetic coverage for scoring of eukaryotic, prokaryotic, and viral genomes. *Molecular Biology and* *Evolution* **38**, 4647–4654.

Steinegger, M., Meier, M., Mirdita, M., Vöhringer, H., Haunsberger, S. J. & Söding, J. (2019). HH-suite3 for fast remote homology detection and deep protein annotation. *BMC Bioinformatics* **20**, 473.
