# Supplementary figures and images for: Lessons from the deep: mechanisms behind diversification of eukaryotic protein complexes
Source: Biol Rev Camb Philos Soc. 2023 Jun 19;98(6):1910–27. doi: 10.1111/brv.12988 (PMC10952624; doi:10.1111/brv.12988)

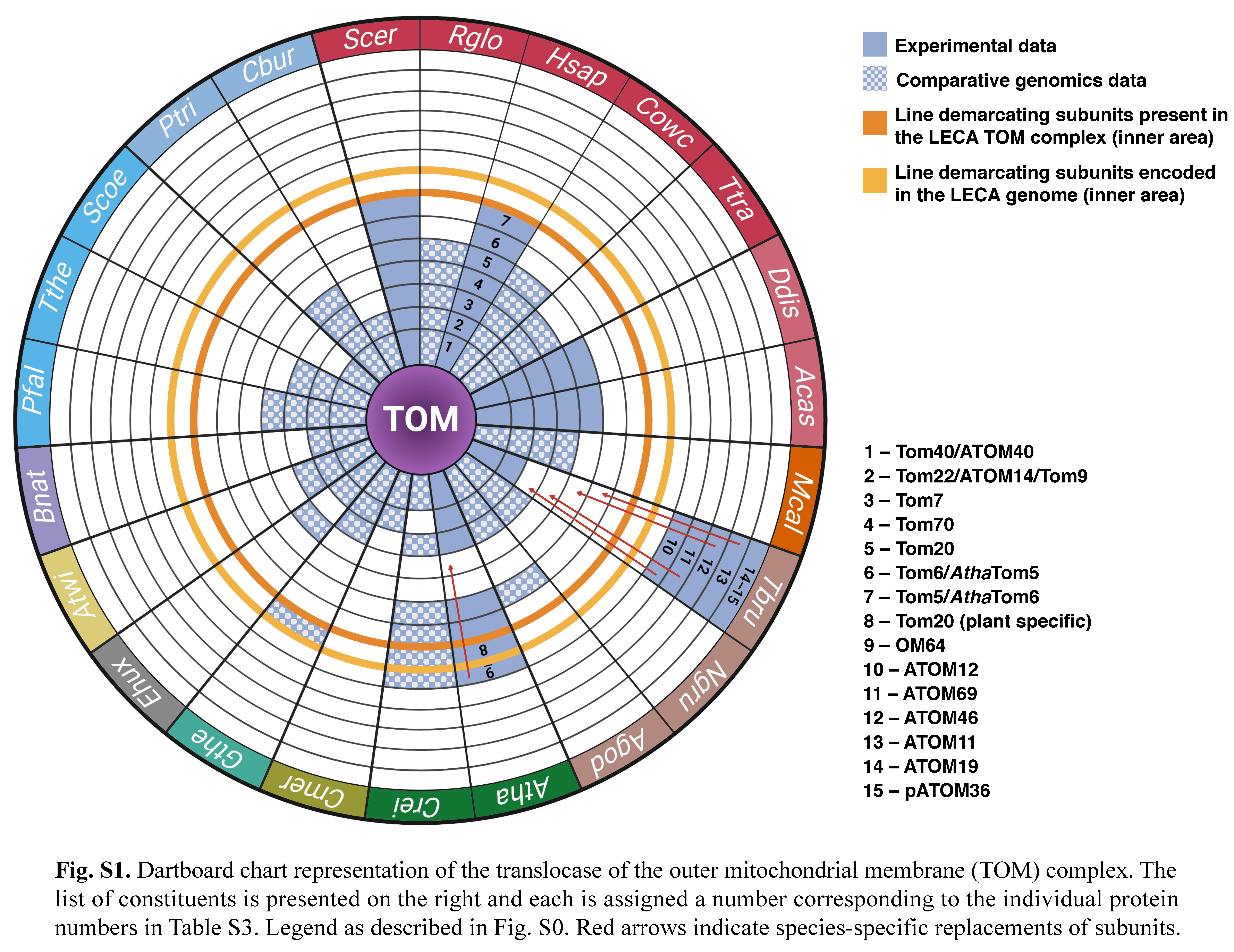

Supplement: Supplementary file 3 — Fig. S1. Dartboard chart representation of the translocase of the outer mitochondrial membrane (TOM) complex. [file BRV-98-1910-s003.tif]

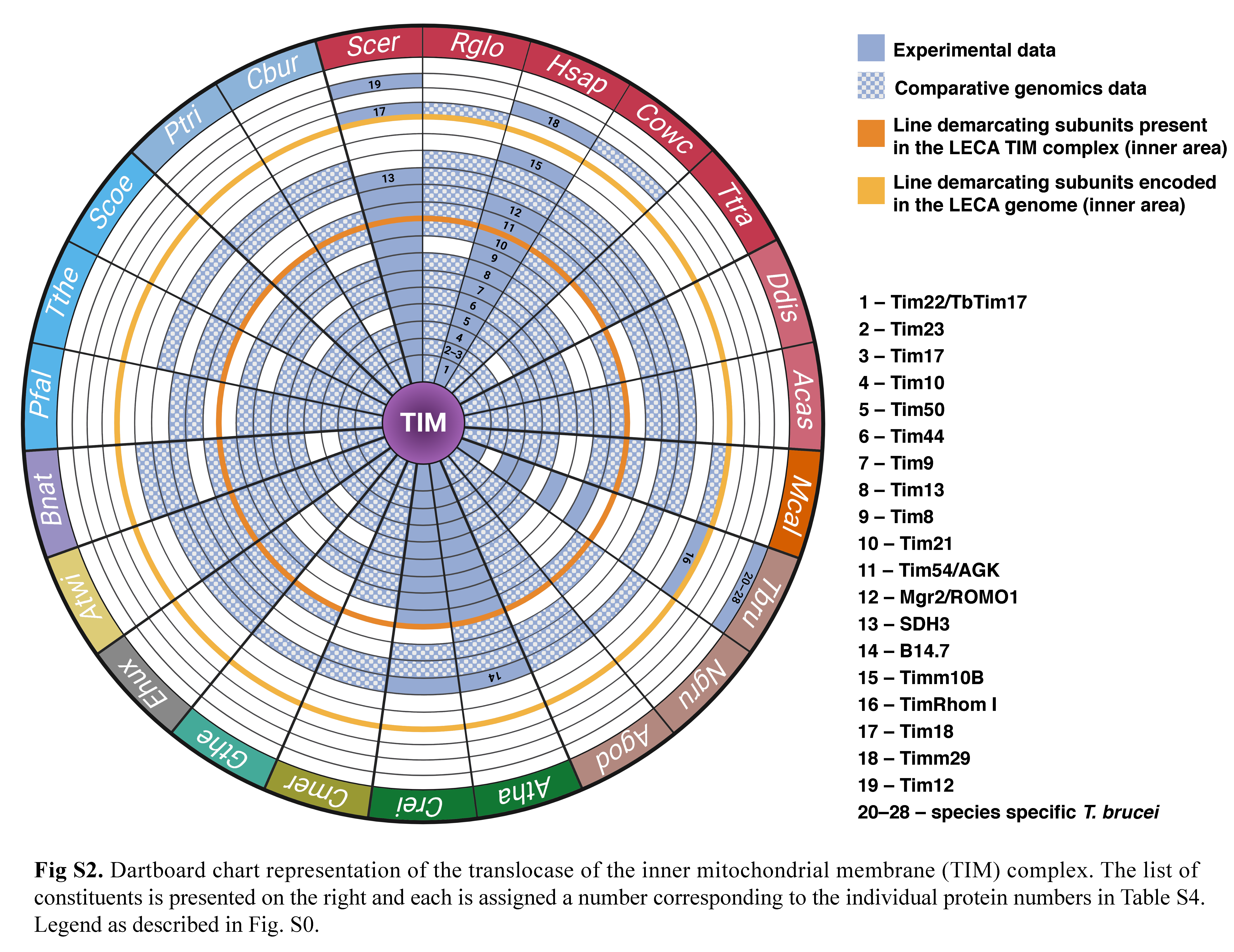

Supplement: Supplementary file 4 — Fig. S2. Dartboard chart representation of the translocase of the inner mitochondrial membrane (TIM) complex. [file BRV-98-1910-s010.tif]

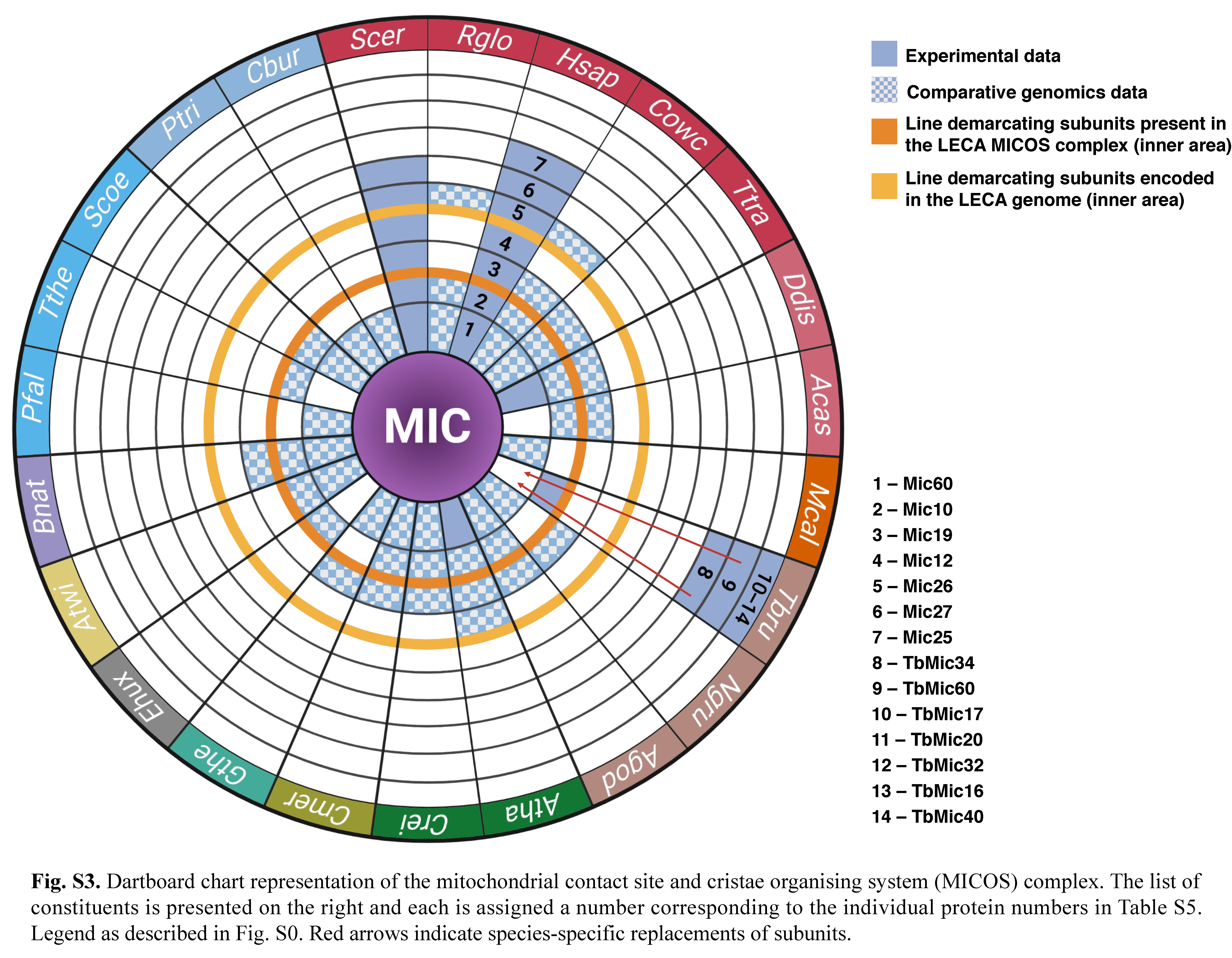

Supplement: Supplementary file 5 — Fig. S3. Dartboard chart representation of the mitochondrial contact site and cristae organising system (MICOS) complex. [file BRV-98-1910-s004.tif]

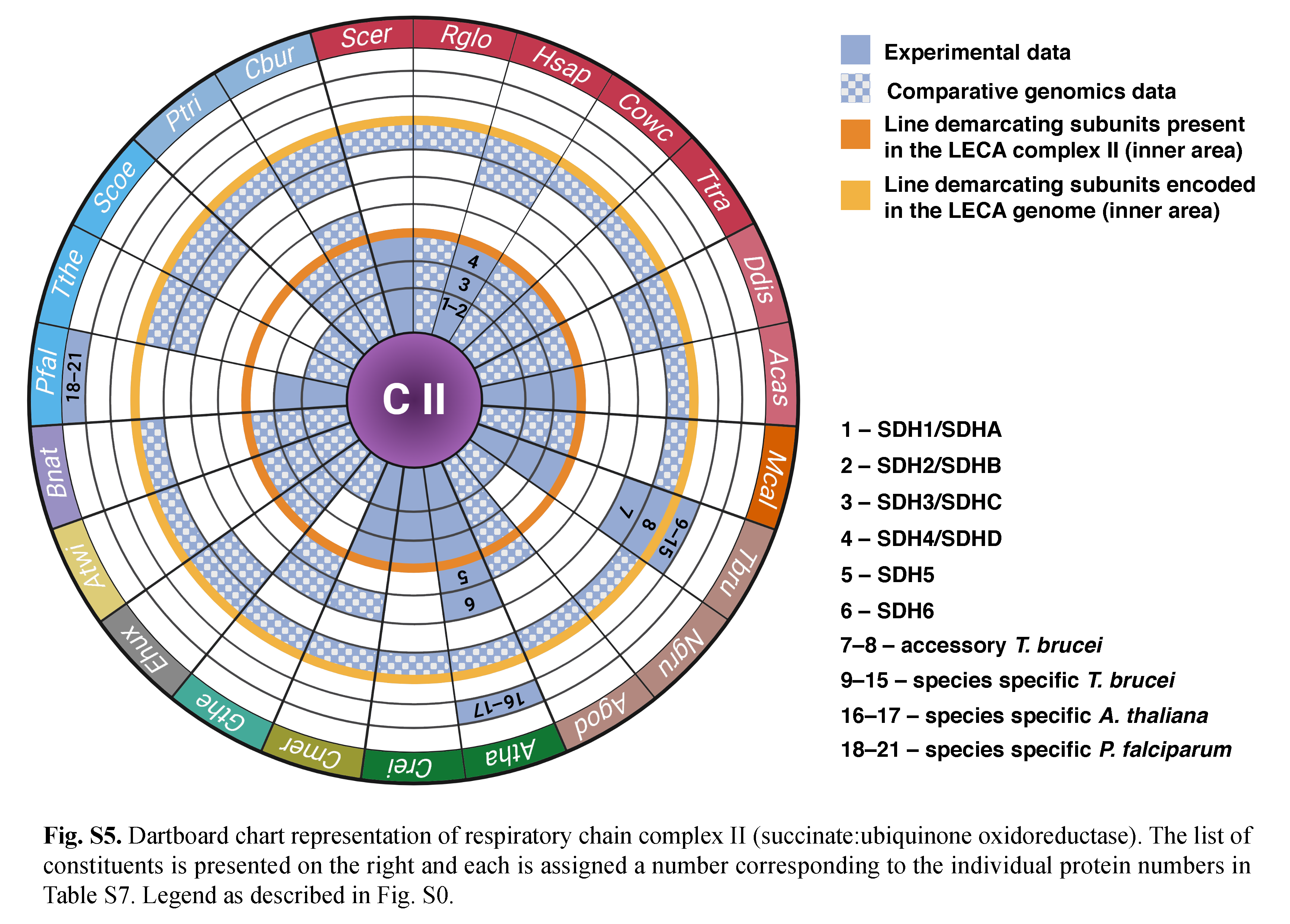

Supplement: Supplementary file 7 — Fig. S5. Dartboard chart representation of respiratory chain complex II (succinate: ubiquinone oxidoreductase). [file BRV-98-1910-s006.tif]

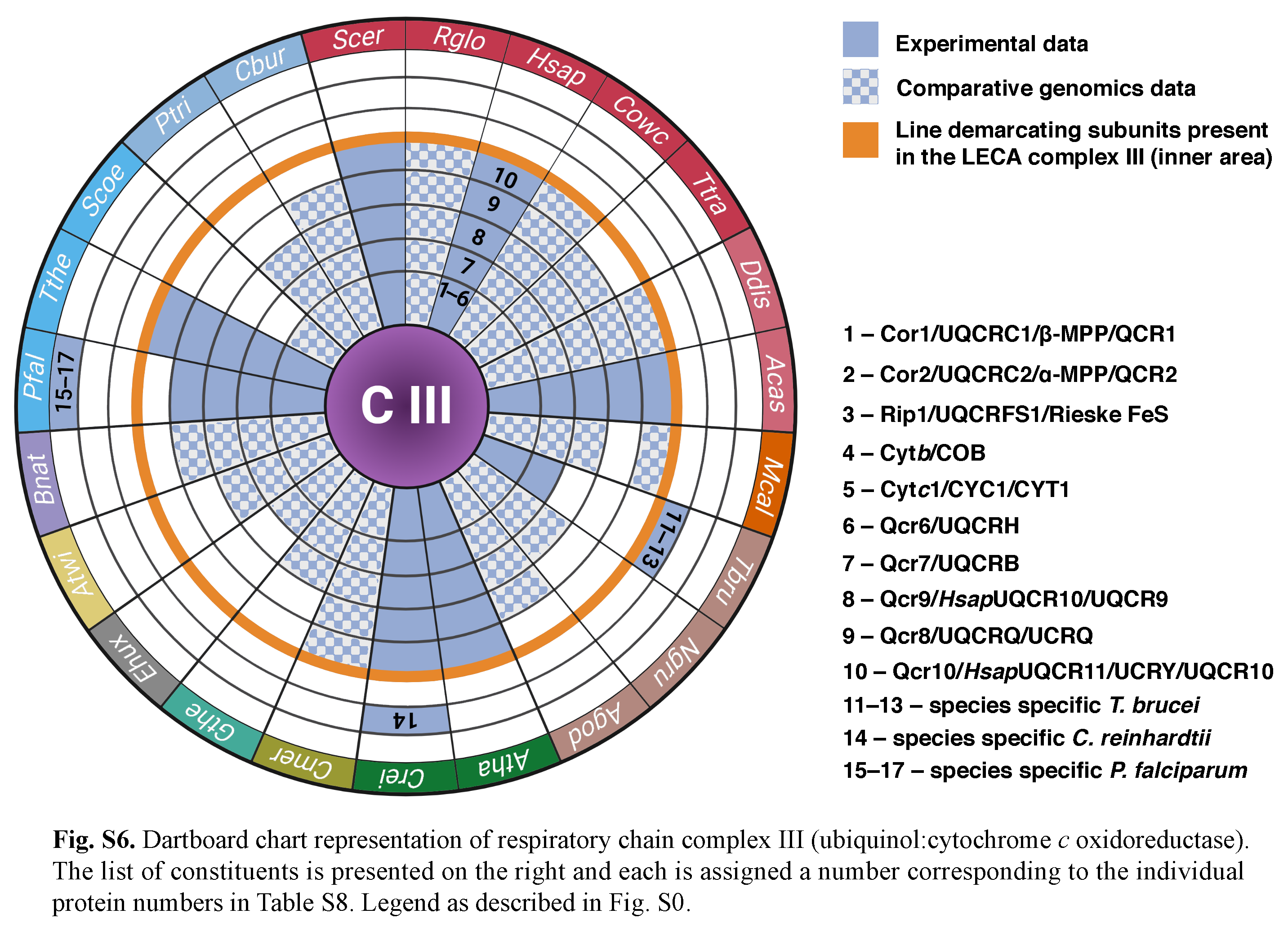

Supplement: Supplementary file 8 — Fig. S6. Dartboard chart representation of respiratory chain complex III (ubiquinol:cytochrome c oxidoreductase). [file BRV-98-1910-s005.tif]

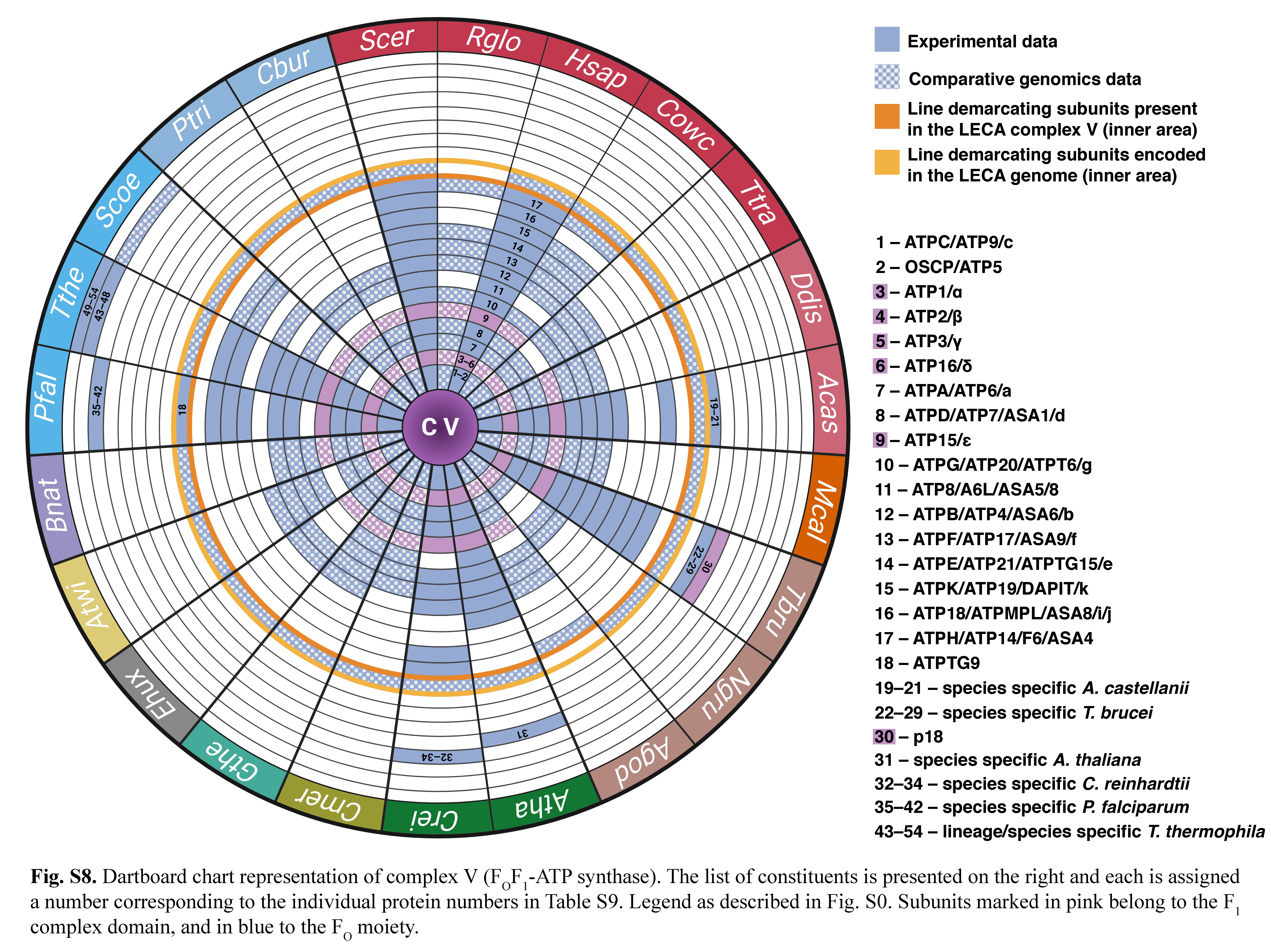

Supplement: Supplementary file 10 — Fig. S8. Dartboard chart representation of complex V (FOF1‐ATP synthase). [file BRV-98-1910-s007.tif]

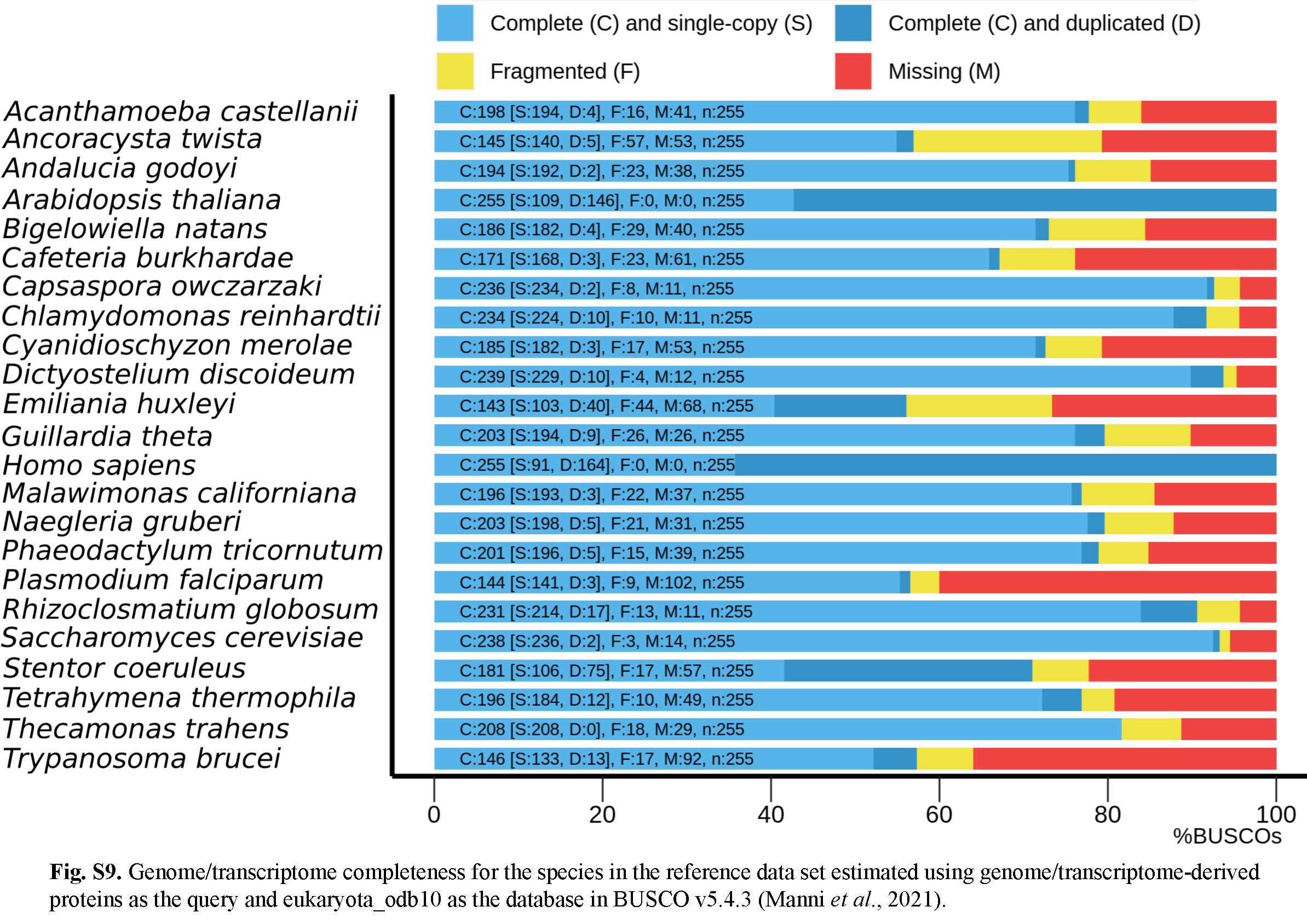

Supplement: Supplementary file 11 — Fig. S9. Genome/transcriptome completeness for the species in the reference data set estimated using genome/transcriptome‐derived proteins as the query and eukaryota_odb10 as the database in BUSCO v5.4.3 (Manni et al., 2021). [file BRV-98-1910-s001.tif]
